# Artificial intelligence technology application and corporate ESG performance—evidence from national pilot zones for artificial intelligence innovation and application

**DOI:** 10.3389/frai.2025.1643684

**Published:** 2025-09-22

**Authors:** Hanjin Xie, Jiayi Luo, Xi Tan

**Affiliations:** ^1^School of Economics and Management, East China Jiaotong University, Nanchang, Jiangxi Province, China; ^2^School of Economics and Management, Wuhan University, Wuhan, Hubei Province, China

**Keywords:** artificial intelligence, corporate ESG performance, difference-in-differences model, sustainable development, data model

## Abstract

This study empirically examines the impact of artificial intelligence (AI) technology on corporate ESG performance using data from Chinese listed companies from 2011 to 2022 and a multi-period difference-in-differences (DID) model. The results reveal that AI significantly enhances overall corporate ESG performance by alleviating financing constraints, promoting green innovation, and strengthening information disclosure. These effects are particularly pronounced in the environmental (E) and governance (G) dimensions. Further analysis indicates that equity concentration, media attention, and data availability positively moderate the relationship between AI adoption and ESG performance. Based on these findings, this study suggests expanding AI application scenarios to facilitate the formulation of more targeted ESG strategies, deepen the integration of AI and ESG practices, and support high-quality economic development. The conclusions provide theoretical and empirical support for technology-driven corporate sustainable transformation.

## 1 Introduction

Over the past two decades, unresolved systemic risks from the 2008 financial crisis, pandemic-induced supply chain disruptions, and prominent social-ecological crises have elevated ESG to a core corporate sustainability metric (Garel and Petit-Romec, 2021; Fang et al., 2023). Investor and corporate focus on ESG has grown, with research exploring its role in long-term value creation, especially in capital markets (Rau and Yu, 2024; Zhang et al., 2022). As digital transformation deepens, AI reshapes corporate operations (e.g., data analysis, environmental monitoring) to boost ESG performance, yet its mechanisms—particularly differential effects on E, S, G dimensions—lack systematic empirical study. Under China’s “Dual Carbon” goals, corporate ESG demands rise, but Chinese firms have long prioritized financial performance with weak non-financial disclosure (Chen, 2022); the endogenous drivers of ESG improvement, and how AI (e.g., easing financing constraints) influences ESG amid its penetration, need investigation.

AI is also transforming the global technological and economic architecture (Parkes and Wellman, 2015; Babina et al., 2024). Machine learning advances have enabled new ESG assessment models (e.g., AI analyzing unstructured data for ESG risks/opportunities) and generative AI for long-term ESG data monitoring (Chen and McCoy, 2024; Wang, 2025)—a global trend, with 83 nations rolling out specialized AI policies for key sectors. The latest AI enhances firms’ data forecasting, optimizes workforce organization, and cuts costs (Agrawal et al., 2019), reshaping industrial operations and creating sustainable development pathways by reconfiguring resource allocation (Dwivedi et al., 2021; Duan et al., 2019; Chen et al., 2024).

As AI-ESG synergy strengthens, the global corporate governance framework transforms, providing new ESG tools—driving firms to integrate AI for better ESG standards. Asif et al. (2023) highlight AI-ESG integration’s strategic value; 134 major economies now have mandatory ESG disclosure rules. Capital market data shows AI-ESG firms improve ratings 2.3x faster and cut financing costs by 147 basis points on average, with AI-driven green financing tied to sustainable goals (Lee et al., 2025). Lin and Zhu (2025) confirm AI’s ESG impact and assessed its societal value in emerging economies like China, noting AI is a core enabler of corporate ESG competitiveness.

Based on this study, we utilize the establishment of China’s National AI Demonstration Zones as a quasi-natural experiment and apply a multi-period difference-in-differences approach to examine the impact of artificial intelligence on corporate ESG performance and its mechanisms, addressing a gap in the existing literature. Compared to prior studies, the marginal contributions of this paper are threefold: First, it introduces a novel technological perspective. While existing research has emphasized traditional factors such as institutional pressure and corporate governance, this study focuses on artificial intelligence as an emerging driver, providing micro-level evidence on how AI shapes corporate ESG performance and enriching the literature at the intersection of technology and sustainable development. Second, it offers more rigorous empirical identification. We not only establish the positive effect of AI on ESG performance but also validate the result through robustness checks such as variable replacement and placebo tests. Furthermore, we identify green technology innovation as a key mechanism and examine contextual moderators, thereby enhancing the theoretical plausibility and empirical granularity of the AI–ESG relationship. Finally, it extends the practical relevance of the findings. The results provide policy insights for scaling up AI pilot zones and designing AI-driven ESG incentives in China. Moreover, the revealed mechanisms—whereby AI improves ESG through innovation and governance pathways—offer a transferable framework for other emerging economies seeking to harness AI for sustainability goals.

The paper is structured as outlined below. Section 2 presents an investigation of the theoretical mechanisms linking AI to organisations’ ESG performance and formulates the relevant research hypotheses. Section 3 delineates the policy identification, utilised data sources, variables, and model formulation for this paper. Section 4 delineates the study of empirical findings, encompassing benchmark regression analysis, robustness assessments, mechanism evaluations, and moderating effects analysis to investigate the potential correlation between AI and corporate ESG performance. Ultimately, Section 6 presents pertinent recommendations derived from the findings of this paper.

## 2 Study of theoretical mechanisms

### 2.1 The influence of artificial intelligence technologies on corporate ESG performance

Scholars have primarily examined the influencing factors of corporate environmental, social, and governance (ESG) performance from both macro and micro perspectives. At the macro level, factors such as economic development, cultural traditions, institutional systems, and legal origins may shape corporate ESG performance (Liang and Renneboog, 2017). At the micro level, elements including institutional ownership and the digital revolution also significantly affect ESG outcomes (Lu et al., 2024). As a core driver of the new technological and industrial revolution, artificial intelligence (AI) is reshaping traditional production and lifestyles while exerting a notable influence on corporate ESG performance (Weng, 2025; Zhang and Yang, 2024). However, existing studies often treat ESG as a monolithic indicator, paying insufficient attention to the distinct mechanisms through which AI affects the environmental (E), social (S), and governance (G) dimensions. A cross-disciplinary perspective integrating AI governance and sustainability economics remains underdeveloped.

To address this gap, this study constructs a dual-path framework of “technological empowerment and governance restructuring” by integrating theories from AI governance and sustainable development economics, aiming to systematically explain the intrinsic mechanisms through which AI enhances ESG performance. The rapidly expanding body of research in finance, particularly on ESG themes and related AI applications, presents challenges for both new researchers and experienced practitioners. We argue that AI technology improves corporate ESG performance across all three dimensions: in the environmental (E) dimension, AI demonstrates significant potential for ecological protection. For instance, AI-driven dynamic resource management platforms can optimize energy topology in real time, substantially reducing carbon emission intensity (Kwok, 2019) and stimulating green technological innovation (Li et al., 2025). Intelligent manufacturing systems enhance energy efficiency and markedly cut industrial carbon emissions (Nishant et al., 2020). More importantly, AI enhances firms’ information acquisition and processing capabilities, driving improvements in energy quality requirements and optimization of energy structures in production processes, thereby facilitating directed technological change.

In the social (S) dimension, AI helps companies accurately identify and respond to the diverse expectations of stakeholders, promoting the implementation of social responsibility practices. Furthermore, by enhancing algorithmic transparency and accountability—a core concern of AI governance—AI strengthens constructive interaction between firms and society. In the governance (G) dimension, AI significantly improves operational efficiency and adaptability through intelligent management of production processes and supply chains (Rammer et al., 2022). Meanwhile, AI-enhanced data integration and analytical capabilities strengthen the quality of ESG disclosures and the effectiveness of compliance monitoring, fundamentally improving corporate governance structures.

Building on this theoretical framework, this study uses the establishment of national AI pilot zones as a quasi-natural experiment to mitigate endogeneity concerns in measuring corporate AI adoption. The empirical results indicate that AI influences the sub-dimensions of ESG through differentiated pathways: the pilot policy notably enhances environmental performance and governance levels primarily by alleviating corporate financing constraints. These findings provide empirical support for the dual-path mechanism of “technological empowerment and governance restructuring,” offering a novel theoretical and empirical understanding of the causal relationship between AI and ESG at the micro level. We therefore posit:

*H1*: Ceteris paribus, AI technology enhances business ESG performance.

### 2.2 Mechanism analysis

#### 2.2.1 Mechanism of financing constraints

Firstly, artificial intelligence (AI) technology can optimize corporate supply chain relationships, facilitating access to more abundant trade credit for enterprises. The increase in trade credit effectively alleviates the financing constraints faced by firms. In recent years, China’s financial system and real economy have exhibited a structural dichotomy characterized by concurrent “capital scarcity” and “funding shortage” (Nikolov et al., 2021). One manifestation of this contradiction at the market level is the relative deficiency in financing channels. While balancing financial stability and innovation, the application of AI can effectively expand the breadth and depth of financial services (Du and Geng, 2024). As an alternative financing method, AI technology provides enterprises with more diversified and accessible financing channels at lower costs compared to traditional avenues, thereby significantly enhancing financing accessibility. Furthermore, the implementation of ESG activities requires substantial, sustained, and stable investments in human and material resources as foundational support (Li et al., 2023). Under severe financing constraints, enterprises often adopt conservative development strategies to mitigate potential financial risks. In capital allocation, firms prioritize maintaining daily operations and achieving short-term profitability objectives, while tending to reduce investments in long-term ESG projects. This tendency inevitably constrains the improvement of corporate ESG performance. Consequently, the application of AI technology expands financing channels and alleviates financing constraints, thereby providing essential financial support for corporate ESG initiatives and ultimately contributing to the enhancement of corporate ESG performance. We therefore posit:

*H2a*: AI technology enhances corporate ESG performance by alleviating financing constraints.

#### 2.2.2 Green technology innovation mechanism

Based on the inherent nature of artificial intelligence (AI) as a technological advancement, its capabilities significantly enhance corporate ESG performance by driving green innovation technologies, revealing a core intermediary mechanism for technology-enabled sustainable development (Dou et al., 2025). Studies indicate that substantive and symbolic green innovations exert differential impacts on enterprises: substantive innovation effectively supports the achievement of sustainable development goals under stringent environmental policies by enhancing corporate environmental adaptability and resource integration efficiency (Zhao et al., 2025). Furthermore, as a pivotal enabling vehicle, AI provides manufacturing firms with green knowledge, information, and technical resources, thereby reconstructing the value network within innovation ecosystems (Huang et al., 2025). It also transforms the entire innovation production process through digitalization and intellectualization (El-Kassar and Singh, 2019), consequently strengthening the synergy between green value creation and ESG practices. In terms of environmental dimensions, AI technology optimizes environmental governance efficacy via real-time production process monitoring, directly contributing to reduced pollutant emissions and resource consumption. Collectively, AI drives profound optimization of corporate environmental (E) and governance (G) dimensions through technological innovation, ultimately enabling systemic enhancement of overall ESG performance (Tian et al., 2025). We therefore posit:

*H2b*: AI technology significantly improves corporate ESG performance by advancing enterprises’ green technology innovation capabilities.

#### 2.2.3 Information disclosure mechanism

ESG serves as a conduit for information transfer, showcasing enterprises’ commendable practices in environmental, social, and governance domains. This fosters social recognition and market trust, alleviates the burdens of support and costs faced by enterprises during production and operations, and ultimately propels their development and value. Regarding the reciprocal relationship between stakeholder value and the insurance function of CSR (Godfrey, 2005), business operators frequently prioritize maximizing financial returns while minimizing social responsibility costs. For instance, some enterprises deliberately diminish the quality of information disclosure (Luo et al., 2017), selectively disclose environmental protection information, and exhibit certain “greenwashing” behaviors (Marquis et al., 2016). The implementation of artificial intelligence technology renders corporate actions recordable and traceable, enhances the transparency of internal information, and diminishes information asymmetry between stakeholders and enterprises. Cao et al. (2023) indicate that this intensifies external supervisory pressure on firms, compelling them to adhere to ESG principles. Consequently, the utilization of AI technology enhances the quality of corporate ESG information and management capabilities, diminishes the costs associated with ESG information management and disclosure, thereby augmenting the intrinsic motivations for corporations to enhance internal governance and more effectively fulfill their social responsibilities. We therefore posit:

*H2c*: AI technology significantly enhances corporate ESG performance by improving the quality and transparency of information disclosure.

### 2.3 Moderating effects

#### 2.3.1 Ownership concentration

Ownership Concentration, a significant aspect of corporate governance outcomes, can influence the internal control mechanism, resource allocation efficiency, and the stability of strategic decisions, thereby profoundly impacting corporate environmental, social, and governance (ESG) factors (Chen et al., 2020). When equity is concentrated among substantial shareholders, these controlling shareholders possess heightened incentives to maximize corporate value, as any increase in corporate value directly correlates with an augmentation of their personal wealth. Furthermore, enhancements in ESG performance not only bolster the firm’s long-term value but also elevate its reputational capital, thereby further amplifying the return on investment for large shareholders (Gedajlovic and Shapiro, 2002). Moreover, elevated equity concentration typically signifies a more stable corporate governance framework, which aids companies in establishing a long-term strategic focus on AI and ESG initiatives. This stability mitigates frequent shifts in decision-making and short-term tendencies arising from equity dispersion, thereby ensuring the ongoing progression of AI-related projects and enhancing corporate ESG performance. Consequently, through the application of AI technology and ESG practices, majority shareholders can harmonize the interests of management, employees, and other internal stakeholders due to their control, mitigate internal conflicts and contradictions, and augment the corporate value of the enterprise (Denis et al., 2003), thereby creating a synergy that collectively enhances enterprise ESG performance. We therefore posit:

*H3a*: Ownership concentration exerts a positive moderating effect on the relationship between AI technology and corporate ESG performance, significantly strengthening their positive association.

#### 2.3.2 Media attention

The media serves as an informal institutional regulator, acting as an observer in the market and facilitating the progression of time. The increase in information transparency aids investors, consumers, regulators, and other stakeholders in accurately evaluating the business conditions and social responsibility performance of enterprises, thereby enabling the formation of more rational market expectations (Dyck et al., 2008). In the realm of AI technology development, public scrutiny profoundly influences business conduct via the reputation mechanism. When a company’s polluting practices are revealed by the media, it typically adversely affects the firm’s environmental acquisition initiatives. Consequently, media scrutiny can serve as a limitation on corporate pollution practices and promote corporate social responsibility (Wang and Zhang, 2021). However, negative media reports can also significantly weaken the positive effects of technological innovation to improve environmental performance and make enterprises face more severe external pressure (Liang et al., 2022). At the same time, media attention can reduce the degree of information asymmetry, bring into play the effectiveness of media governance, and urge enterprises to fulfill their social responsibilities (He and Li, 2024). Therefore, the application of AI technology is more likely to bring about an improvement in ESG performance in firms with higher media attention. We therefore posit:

*H3b*: Media attention exerts a positive moderating effect on the relationship between AI technology and corporate ESG performance, thereby strengthening their association.

#### 2.3.3 Data elements

Data elements serve as the fundamental basis for enterprise decision-making, optimizing business processes and improving production, operational, and managerial efficiency. Pete’s innovation theory posits that the sustained economic development fundamentally arises from the reintegration and efficient allocation of production factors. This theoretical framework demonstrates that the judicious utilization of public data has effectively broadened the parameters of the current production function, equipping enterprises with both prospective and empirical research instruments and a foundation for decision-making in their innovative endeavors (Einav and Levin, 2014). Motivated by data productivity, the corporate R&D model, organizational outcomes, and resource methodologies evolve, hence continuously expanding the value creation trajectory (Ciriello et al., 2018), which encourages organizations to enhance ESG practices in innovation. Simultaneously, as AI technology enhances enterprise ESG practices, the richness, diversity, and advanced decision-making capabilities of data pieces can be fully realized. They can enhance corporate efficiency immediately (Müller et al., 2018) and assist in identifying and implementing ideal manufacturing methods through precise data forecasting, so substantially elevating product quality (Brekke et al., 2003). Consequently, data elements provide efficient responses via an intelligent AI decision-making system, thereby optimizing resource allocation and enhancing overall ESG performance. We therefore posit:

*H3c*: Data elements exerts a positive moderating effect on the relationship between AI technology and corporate ESG performance, thereby strengthening their association.

## 3 Recognition strategy and research design

### 3.1 Identification strategy

Measuring AI technology is a critical problem in the investigation of AI technology and business ESG performance. China’s AI development adheres to the strategic principle of “empowering the real economy and facilitating social development,” and has established a distinctive research and development structure and application ecosystem with Chinese features. National Pilot Zones for Artificial Intelligence Innovation and Application (hereinafter “Pilot Zones”) are established by the Chinese government to advance the development and utilization of AI technology, aiming to facilitate the profound integration of AI with economic and social development through policy guidance and support. The establishment of these pilot zones prioritizes the demonstration of AI technology, policy experimentation, and social experimentation, aiming to address significant challenges in AI technologization and industrialization. It seeks to innovate systems and mechanisms, enhance the integration of industry, academia, research, and application, foster the convergence of science and technology, industry, and finance, and create a favorable ecosystem for AI development. Simultaneously, it offers a valuable study perspective for this paper’s investigation.

In May 2019, the Ministry of Industry and Information Technology sanctioned the establishment of the nation’s inaugural pilot zone for artificial intelligence innovation and application in the Pudong New Area of Shanghai. This initiative aims to systematically develop the national experimental zone for the innovative advancement of a new generation of artificial intelligence, maximize the role of key stakeholders, and investigate novel pathways for the profound integration of artificial intelligence with economic and social development. In October 2019, the MIIT further facilitated the establishment of the inaugural cohort of AI innovation and application pilot zones in Jinan-Qingdao and Shenzhen. In February 2021, the finalized list of the second cohort of national AI innovation and application pilot zones included Beijing, Tianjin (Binhai New Area), Hangzhou, Guangzhou, and Chengdu. In October 2022, the MIIT officially communicated with the People’s Governments of Jiangsu, Hunan, and Hubei provinces to endorse the establishment of National Pilot Zones for AI Innovation and Application in Nanjing, Wuhan, and Changsha. At the conclusion of 2022, the quantity of pilot zones attained 12. The pilot zone legislation creates favorable conditions for the implementation of AI technology to achieve green transformation and offers a chance for this paper’s research. This study will utilize the establishment of national pilot zones for AI invention and application as a quasi-natural experiment.

**Figure 1:**
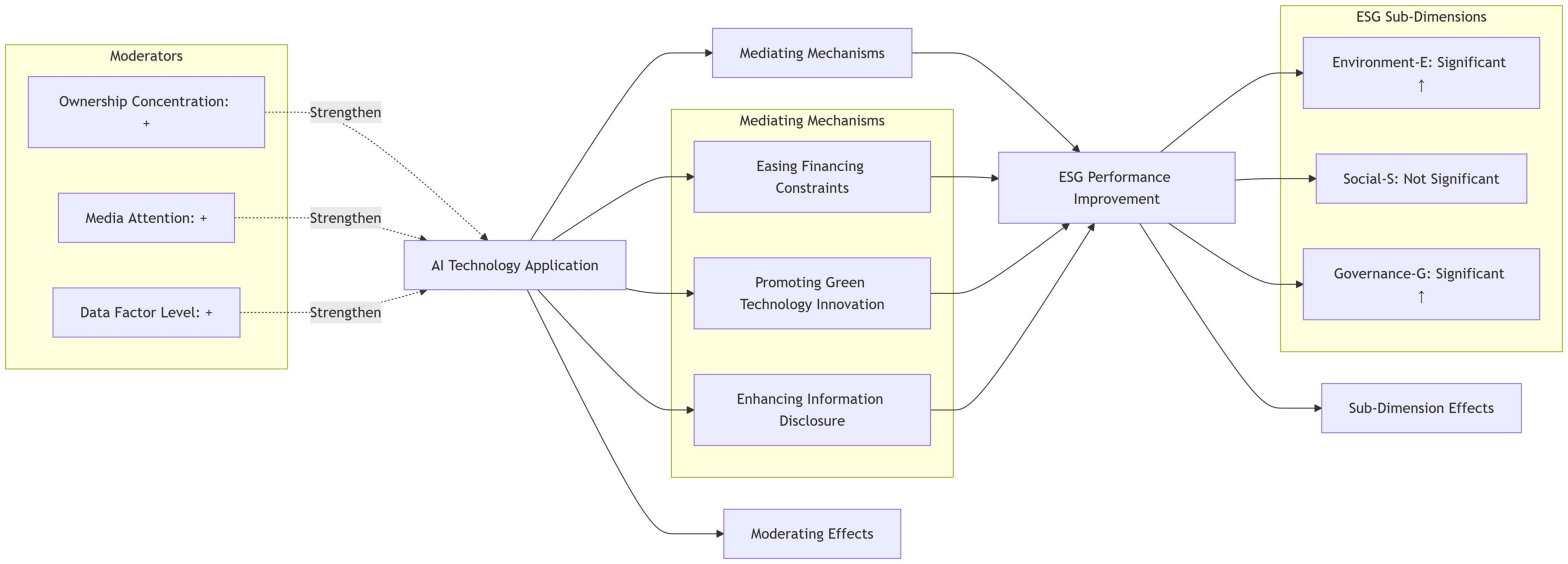
Research framework.

The specific research framework is shown in Figure 1.

### 3.2 Research design

#### 3.2.1 Configuration of the model

This paper will utilize the National Pilot Zone of Artificial Intelligence Innovation and Application as a quasi-natural experiment to empirically examine the relationship between the influence of AI technology and the ESG performance of enterprises, employing a multi-period double-difference model. The regression model is structured as follows:


(1) $$\mathrm{ES}G_{i.t}=\beta_{0}+\beta_{1}AI_{i,t}+\sum_{j}\;\beta_{j}\text{Control}s_{j,i,t}+v_{i}+u_{t}+\varepsilon_{i,t}$$

In this research, we utilize $AI_{i,t}$ to represent “treat×post “within the multi-period Difference-in-Differences model about the national AI innovation and application pilot zone policy. Treat indicates whether the enterprise is situated in a pilot zone, assigned a value of 1 if it is, and 0 if it is not; Post is a temporal dummy variable, valued at 1 for the period of establishment and the subsequent years, and 0 otherwise. The pilot zones in Pudong New Area, Shanghai, Shenzhen, Jinan, and Qingdao were developed in 2019. Pilot zones will be built in Beijing, Tianjin Binhai New Area, Hangzhou, Guangzhou, and Chengdu in 2021. Pilot zones in Nanjing, Wuhan, and Changsha will be constructed in 2022. Due to the two-way fixed-effects model for individuals and years, only $AI_{i,t}$ is incorporated into the model to circumvent multicollinearity issues. $\mathrm{ES}G_{i.t}$ denotes the ESG performance of firm i at time t. Controls refer to the control variables. “Controls “constitute the array of control variables; j signifies the quantity of control variables; “v” defines firm-level fixed effects; “u” indicates year fixed effects; “*ε*” represents the stochastic error term; “i,t “refers to firm and time, respectively.

#### 3.2.2 Variable definition

##### 3.2.2.1 Explanatory variables

The primary explanatory variable of this study is AI technology, quantified by the national AI innovation and application pilot zone policy $AI_{i,t}$ . The pilot zone policy, being an external shock, effectively mitigates the endogeneity issue arising from autonomous selection. Selecting firm-level AI indicators will result in a significant endogeneity issue concerning businesses’ ESG performance, hindering our ability to effectively ascertain the causal relationship between AI progress and firms’ ESG performance.

##### 3.2.2.2 Explained variables

The independent variable of this study is corporate ESG performance ( $\mathrm{ES}G_{i.t}$ ). The explained variable in this study is corporate ESG performance (ESG). Currently, multiple ESG rating systems exist both domestically and internationally. To ensure the scientific rigor and robustness of the research conclusions, the selection of a specific rating system for the core model requires solid theoretical justification. This paper primarily employs the HuaZheng ESG rating score as a proxy variable for corporate ESG performance, for the following reasons: First, considering data availability and coverage, the HuaZheng ESG rating comprehensively covers all A-share listed companies, effectively mitigating sample selection bias caused by incomplete rating coverage and thereby ensuring the generalizability of the research findings. Second, given its advantage in local context adaptability, compared to international rating agencies, the HuaZheng ESG evaluation system incorporates not only internationally accepted criteria but also fully accounts for China-specific institutional backgrounds and policy orientations (such as the “Dual Carbon” goals and the green development strategy). Its indicator framework is thus better designed to accurately measure the true ESG performance of Chinese enterprises. The system encompasses 14 thematic categories, 26 tertiary indicators, and over 130 underlying data points, constituting a comprehensive structure. Based on this, the study uses the HuaZheng ESG comprehensive score as a quantitative measure of corporate ESG performance (ESG). A higher value indicates a better ESG rating. Furthermore, we fully acknowledge the potential measurement errors associated with relying on a single ESG metric. Therefore, in the robustness test section, Bloomberg ESG scores and numerically converted HuaZheng ESG rating data are adopted as alternative measures to cross-validate the reliability of the core findings.

##### 3.2.2.3 Control variables

To govern supplementary factors affecting firms’ ESG performance. According to the existing literature, the following control variables are identified: Firm size (Size) is measured as the logarithmic value of total assets; the gearing ratio (Lev) is defined as the proportion of total liabilities to total assets; return on total assets (ROA) is computed as the ratio of return on investment to total investment; the cash flow ratio (Cash) is established as the ratio of net operating cash flow to total assets. Tobin’s Q value (TobinQ) is the ratio of a company’s stock market value to the cost of creating a new enterprise; institutional investors’ shareholding (Inst) denotes the percentage of shares owned by institutional investors; the employee count (Employee) is represented by the logarithm of the total number of employees; management’s age (Age) is determined by the age of the management team; and the existence of an overseas background among directors and supervisors (Oversea_tmt) is indicated by a value of 1 if such experience is present, and 0 if absent.

#### 3.2.3 Data source and processing

This study selects A-share listed businesses in Shanghai and Shenzhen from 2011 to 2022 as the initial sample, excluding those with special treatment (ST and ST*), resulting in 18,667 firm-year observations after addressing missing data. This study employs 1 and 99% two-sided shrinkage on continuous variables to mitigate the influence of data extremes on research outcomes. Information regarding firms’ ESG ratings is sourced from the Wind database, while data on firms’ green patents is obtained from the China Research Data Service Platform (CNRDS). The remaining financial metrics are sourced from the CSMAR database. Table 1 presents the descriptive statistics for each variable.

**Table 1:** Descriptive statistics.

Variable	*N*	Mean	SD	Min	Max
ESG	18,667	73.06	5.60	38.92	92.93
AI	18,667	0.09	0.28	0	1
Size	18,667	22.52	1.37	17.87	28.64
Lev	18,667	0.36	0.15	0.01	1.93
Roa	18,667	0.11	0.26	−2.65	4.39
Cash	18,667	0.05	0.07	−0.76	1.17
TobinQ	18,667	1.96	1.61	0.64	76.82
Inst	18,667	45.90	24.74	0	157.10
Employee	18,667	7.93	1.30	2.30	13.25
Age	18,667	49.57	3.20	35.6	62.88
Oversea_tmt	18,667	0.54	0.50	0	1

## 4 Empirical results and analysis

### 4.1 Multicollinearity test

To mitigate potential distortions in regression results caused by multicollinearity among explanatory variables, this study employs variance inflation factors (VIF) alongside correlation matrices for diagnostic assessment. By incorporating the core independent variable (AI technology adoption) and all control variables into a unified regression model, we calculated VIF values for each variable. As presented in Table 2, all VIF values remain below the critical threshold of 3.0, with a mean VIF value of 1.47—substantially lower than the conventional benchmark of 10. These results collectively confirm the absence of severe multicollinearity issues, thereby ensuring the validity and robustness of our regression estimates.

**Table 2:** Multicollinearity test.

Variable	(1)	(2)
	VIF	1/VIF
AI	1.04	0.569***
Size	3.10	1.041***
Lev	1.37	−9.487***
ROA	1.00	−0.000***
Cash	1.09	−1.214*
Tobinq	1.16	−0.024
inst	1.34	0.003
Employee	2.34	0.427
Oversea	1.03	0.973
Age	1.26	0.794
Mean VIF	1.47	

The standard errors in brackets are ***p < 0.01, **p < 0.05, and *p < 0.1.

### 4.2 Benchmark regression results

Table 3 presents the baseline regression results analyzing the impact of Artificial Intelligence (AI) technology on corporate ESG performance. Column (1) reports the results without control variables, while Column (2) builds upon Column (1) by incorporating control variables. The results indicate that the coefficient on AI remains significantly positive regardless of the inclusion of control variables. This suggests that AI technology, potentially through big data analytics and algorithmic optimization, enhances the precision of decision-making and execution efficiency concerning environmental management, social responsibility fulfillment, and governance effectiveness. Concurrently, it reduces the marginal costs associated with corporate ESG implementation. From a long-term development perspective, AI technology holds the potential to emerge as a pivotal driving force for improving corporate ESG performance. Therefore, the significantly positive impact of AI on ESG performance can be attributed to the combined effects of technological empowerment, cost optimization, and strategic alignment. This finding provides empirical evidence for understanding how digital transformation fosters corporate sustainable development and supports research Hypothesis H1.

**Table 3:** Fundamental regression analysis.

Variable	(1)	(2)	(3)	(4)
	ESG	ESG	ESG	ESG
AI	0.579^***^	0.569^***^	5.918^***^	5.524^***^
	(0.206)	(0.191)	(1.891)	(1.779)
Size		1.041^***^		1.045^***^
		(0.163)		(0.649)
Lev		−9.487^***^		−5.798^***^
		(0.696)		(0.648)
ROA		−0.000^***^		−0.000^***^
		(0.000)		(0.004)
Cash		−1.214^*^		−1.420^**^
		(0.620)		(0.632)
Tobinq		−0.024		0.039
		(0.056)		(0.045)
inst		0.003		0.001
		(0.004)		(0.004)
Employee		0.908^***^		0.859^***^
		(0.139)		(0.140)
Oversea		−0.185		−0.141
		(0.123)		(0.126)
Age		0.095^***^		0.095^***^
		(0.030)		(0.030)
Constant	73.007^***^	41.072^***^	73.004^***^	40.538^***^
	(0.018)	(3.407)	(0.019)	(3.371)
predefined	No	No	Yes	Yes
Year	Yes	Yes	Yes	Yes
Id	Yes	Yes	Yes	Yes
*N*	18,667	18,667	17,806	17,485
*R* ^2^	0.537	0.566	0.493	0.523

The standard errors in brackets are ***p < 0.01, **p < 0.05, and *p < 0.1.

To more accurately ascertain the causal impact of AI technology on enterprises’ ESG performance, predetermined variables influencing the designation of a region as an AI pilot zone are incorporated into the regression equation. Initially, while selecting a pilot zone, the government may evaluate the region’s scientific and educational resources, industrial foundation, infrastructure, degree of internationalization, amount of policy support, and economic development status. The variables influencing policy formulation are commonly termed predefined variables. This paper incorporates a cross-multiplier term into the regression equation, which multiplies the values of the relevant variables from the year preceding the policy’s implementation with a dummy variable indicating the year it became an innovative zone (Post). Specifically, scientific and educational resources are quantified by the quantity of effective invention patents in artificial intelligence held by research institutions and high-tech companies; The degree of industrial agglomeration indicates the extent to which firms have achieved the aggregation effect within the AI industry, quantified by the number of AI enterprises in prefecture-level cities; Infrastructure indicates the region’s foundational conditions for AI development, assessed through six indicators related to investments in both general and digital infrastructure; the degree of internationalization, which enables a region to assimilate advanced AI technologies from abroad, is quantified by the total volume of imports and exports relative to the regional GDP. Policy support is crucial, as local governments prioritize AI development, explicit policy and financial backing are essential criteria for becoming a pilot zone, assessed by the ratio of regional imports and exports to regional gross domestic product (GDP), while economic development is gauged by the level of regional GDP. Table 3 presents the regression outcomes incorporating the predetermined variables; column (3) displays the results with only the predetermined variables, while column (4) exhibits the results with both the control and predetermined variables included. The findings demonstrate that AI technology continues to play a substantial role in enhancing organizations’ ESG performance when considering the influence of preceding variables.

### 4.3 Robustness check

#### 4.3.1 Parallel trend analysis

**Figure 2:**
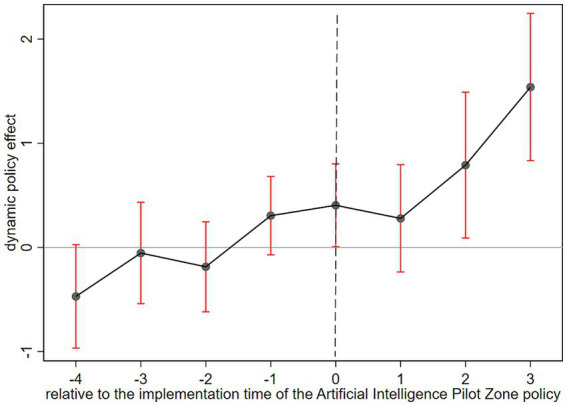
Parallel trend analysis.

The validity of the double-difference model for assessing the impact of policy implementation depends on the experimental and control groups meeting the parallel trend criterion prior to the policy’s enactment; specifically, the trajectory of ESG performance changes in enterprises within the pilot zone must align with those outside the pilot zone before the intervention. This work employs the concept proposed by Beck et al. (2010) to analyze the dynamic influence of AI technology on the ESG impact of organizations, utilizing the event study approach for the parallel trend test. The specific model description is given by Equation 2:


(2) $$\mathrm{ES}G_{\mathrm{it}}=\alpha +\sum_{k=-4,k\neq -1}^{3}\beta_{k}+\gamma X_{\mathrm{it}-1}+v_{i}+u_{t}+\varepsilon_{i,t}$$

where $D_{i,t+k}\;$ is a set of dummy variables that takes the value of 1 if the city where firm i is located is listed as a national pilot zone for AI innovation and application in yeart+k, and vice versa, takes the value of 0. The rest of the variables have the same sign as in Equation 1. This paper focuses on the coefficient β_k in this equation, which reflects the difference in ESG performance of listed firms within the pilot zone cities and non-pilot zone cities in the kth year before the pilot implementation of the national pilot zone for AI innovation and application policy. This paper uses the period immediately before the implementation of the National Pilot Zone for AI Innovation and Application policy as the baseline for estimating dynamic effects. As shown on the left side of Figure 2, the estimated coefficients and their 95% confidence intervals indicate that all pre-treatment period coefficients are statistically insignificant. This suggests no significant pre-existing trend differences in ESG performance between the treatment and control groups prior to the policy intervention, thus satisfying the parallel trends assumption. After the policy implementation, the regression coefficients become significantly positive and exhibit a sustained upward trend, implying that AI technology may have a persistent and positive enhancing effect on corporate ESG performance.

#### 4.3.2 Placebo testing

**Figure 3:**
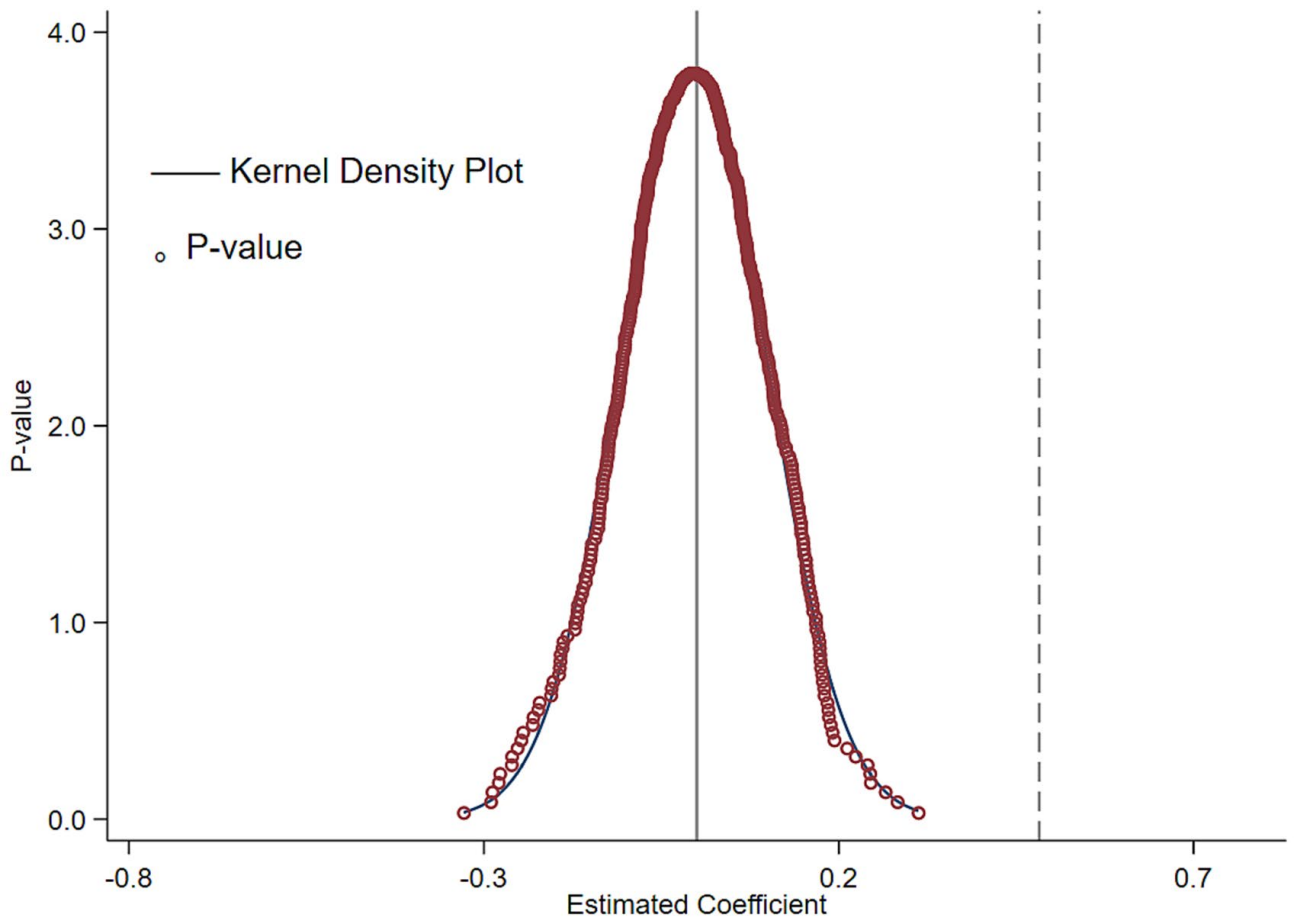
Placebo testing.

Another hypothesis about the construction of a multi-temporal double-difference model is that unobservable factors influence the selection of national AI innovation and application pilot zones in a non-random manner. This research employs a placebo test to exclude the influence of external random influences on its conclusions. This paper randomly selects samples from the experimental group and the timing of policy implementation, reconstructs the policy dummy variables, and substitutes the reconstructed dummy variables “ ${\text{treat}}_{i}\times {\text{time}}_{t}$ ” into the baseline regression model (1). This process is repeated 500 times, after which the regression coefficients of “ ${\text{treat}}_{i}\times {\text{time}}_{t}\;$ ” from the 500 regression iterations are plotted. Since the experimental group samples and the establishment time of pilot zones are randomly selected, the falsely constructed experimental groups from random simulations should not exhibit statistical significance. If the distribution of estimated coefficients under random treatment clusters around 0, it implies that the false policy variable has no significant impact on corporate ESG performance—indicating that the impact effect observed in the baseline regression analysis of this study is indeed driven by the National AI Innovation and Application Pilot Zone Policy. As can be seen from the estimated coefficient plot in Figure 3, the false regression coefficients are concentrated around 0, while the baseline regression coefficient (0.569) is significantly different from 0 and far from the distribution of false coefficients. To a certain extent, this demonstrates that the conclusions of the baseline regression in this study are not caused by accidental observed factors.

#### 4.3.3 Replacement of explanatory variables

This research aims to substitute the CSI ESG scores with the ESG scores from the Bloomberg system to assess the robustness of the estimation results, given that the outcomes may be influenced by the chosen variable measures and rating methodologies. Concurrently, the CSI scores are allocated a rating from 1 to 9 for the nine ESG ratings based on various metrics, as referenced by Lins et al. (2017), with higher values signifying superior ratings, hence functioning as the secondary replacement variable. The estimation findings are presented in Table 4, and they remain significant following the substitution of the explanatory factors. It signifies that the enabling influence of AI technology on companies’ ESG performance persists.

**Table 4:** Replacement of explanatory variables.

Variable	(1)	(2)	(3)	(4)
	ESG rating	ESG rating	Bloomberg	Bloomberg
AI	0.130^***^	0.134^***^	0.862^*^	0.887^**^
	(0.041)	(0.038)	(0.450)	(0.450)
Constant	4.107^***^	−3.240^***^	23.799^***^	28.032^***^
	(0.004)	(0.605)	(0.038)	(3.376)
Control	No	Yes	No	Yes
Year	Yes	Yes	Yes	Yes
Id	Yes	Yes	Yes	Yes
*N*	18,287	17,964	8,105	8,007
*R* ^2^	0.514	0.537	0.831	0.832

The standard errors in brackets are ***p < 0.01, **p < 0.05, and *p < 0.1.

#### 4.3.4 Adjustment policy window

The research sample’s duration in this paper is considerable, and by reducing the sample period, the temporal stability of the data can be evaluated to ascertain their trustworthiness. The initial pilot zones were established in 2019, and to enhance the reliability of the findings, this study narrows the sample period to 2015–2022, ensuring policy consistency. The robustness findings in columns (1) and (2) of Table 5 indicate that the regression coefficients of AI continue to be significant at the 1% level following the reduction of the sample duration.

**Table 5:** Adjusting policy windows and removing other policy distractions.

Variable	(1)	(2)	(3)	(4)	(5)
	ESG	ESG	lowco2	Greenfinance	Multiple policy shocks
AI	0.130^***^	0.134^***^	0.604^***^	0.634^***^	0.638^***^
	(0.041)	(0.038)	(0.190)	(0.189)	(0.189)
lowco2			−0.613^**^		−0.542^**^
			(0.241)		(0.243)
greenfinance				−0.413^**^	−0.376^*^
				(0.205)	(0.206)
constant	4.107^***^	−3.240^***^	35.036^***^	34.616^***^	34.762^***^
	(0.004)	(0.605)	(3.273)	(3.282)	(3.275)
Control	No	Yes	Yes	Yes	Yes
Year	Yes	Yes	Yes	Yes	Yes
Id	Yes	Yes	Yes	Yes	Yes
*N*	18,287	17,964	17,964	17,964	17,964.
*R* ^2^	0.514	0.537	0.563	0.563	0.563

The standard errors in brackets are ***p < 0.01, **p < 0.05, and *p < 0.1.

#### 4.3.5 Exclusion of other policy interferences

Failing to account for concurrent policies during the study period may lead to biased estimates of the treatment effect. To isolate the impact of the National AI Demonstration Zones on corporate ESG performance, it is essential to control for other relevant policy interventions. Notably, the Green Finance Reform Pilot was launched in 2017, and the second and third groups of Low-Carbon City Pilots were also announced in 2017—both overlapping with the policy period under examination. These initiatives could independently influence firms’ ESG performance, potentially confounding the causal interpretation of the AI zone policy.

To address this, we include dummy variables for the green finance reform (greenfinance) and low-carbon city pilot (lowco2) policies in our baseline regression. As shown in columns (3) to (5) of Table 5, the estimated coefficient of the AI policy remains positive and statistically significant at the 1% level, whether we control for each policy separately or include both simultaneously. These results reinforce the robustness and validity of our main findings, confirming that the estimated effect of the AI zones on ESG is not driven by other concurrent policy shocks.

#### 4.3.6 Excluding the 2022 cohort of pilot zones

To further verify the reliability of our findings while addressing the staggered difference-in-differences model’s sensitivity to policy timing, this section conducts robustness checks by excluding enterprise samples from national AI pilot zones newly established in 2022. Specifically, the original sample contained observations from three pilot zones approved in 2022 (Nanjing, Changsha, Wuhan). Given their short policy exposure periods and limited post-treatment data, these samples might potentially affect estimation stability. Consequently, we remove all 2022 pilot observations (Nanjing, Changsha, Wuhan) and retain only zones approved in 2021 or earlier for re-estimation. Results in Table 6 demonstrate that after excluding 2022 samples, the core explanatory variable (interaction term of policy dummy and time dummies) maintains identical coefficient sign, statistical significance, and comparable effect magnitude relative to baseline regressions. This consistency confirms that the impact of AI pilot zones on corporate ESG performance remains materially unchanged, indicating our core conclusions persist robustly without late-cohort samples and are not significantly distorted by recently implemented pilots.

**Table 6:** Excluding the 2022 cohort of pilot zones.

Variable	(1)	(2)
	ESG	ESG
AI	0.621***	0.608***
	(0.214)	(0.198)
Constant	72.998***	40.414***
	(0.019)	(3.440)
Control	No	Yes
Year	Yes	Yes
Id	Yes	Yes
*N*	17,452	17,136
*R* ^2^	0.491	0.537

The standard errors in brackets are ***p < 0.01, **p < 0.05, and *p < 0.1.

In summary, the stable regression results after excluding short-exposure samples further validate AI development’s promoting effect on corporate ESG performance, reinforcing the empirical credibility of our baseline findings.

#### 4.3.7 Propensity score matching—difference in differences (PSM-DID)

**Figure 4:**
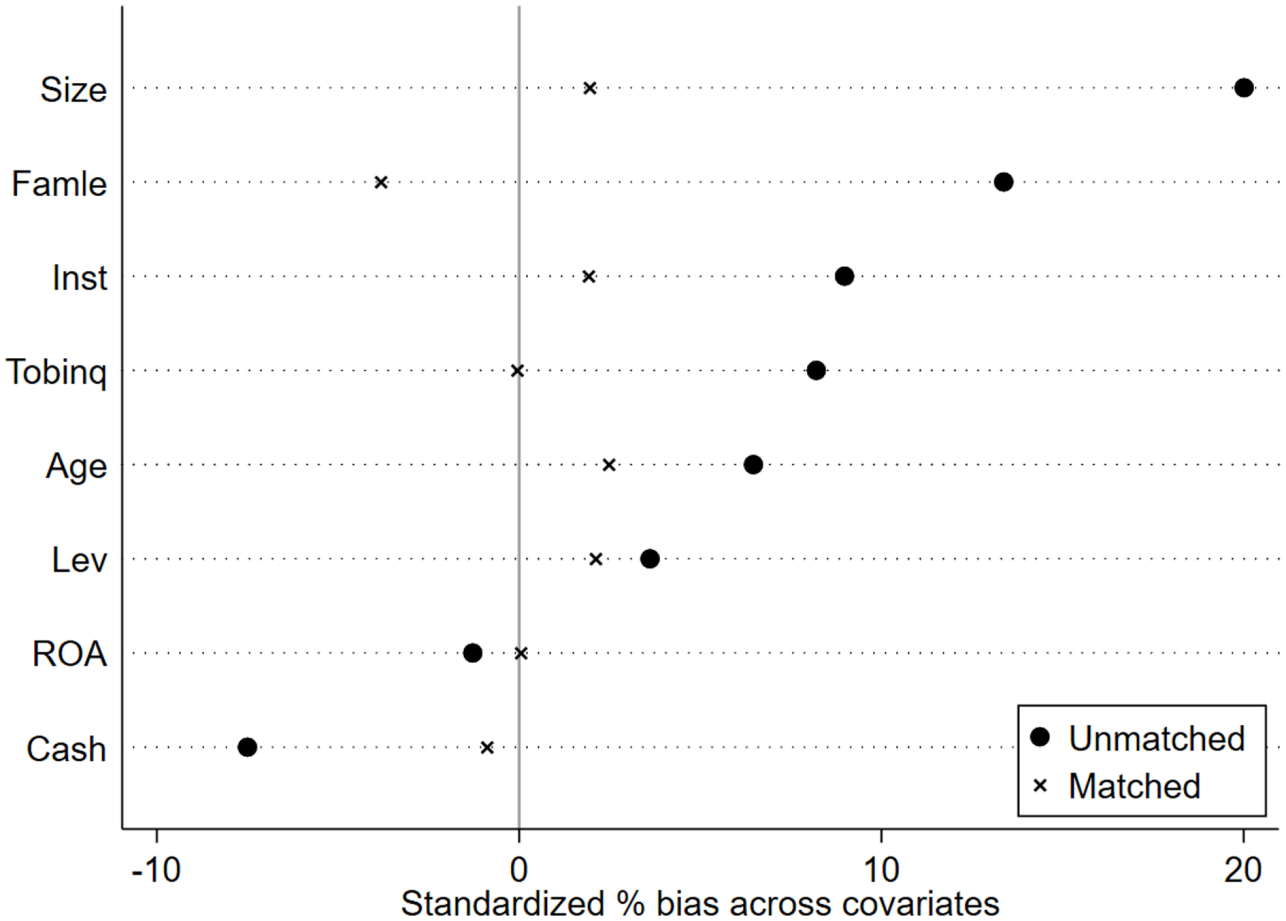
PSM Balance Test Chart-Mixed match nearest neighbor 1:1.

**Figure 5:**
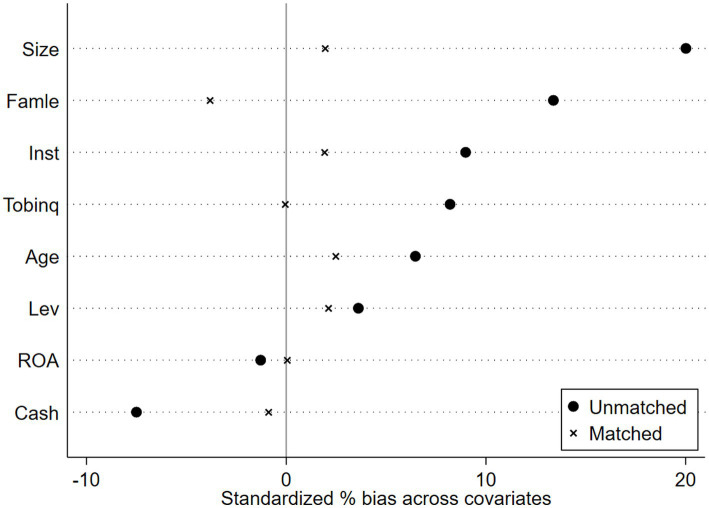
PSM Balance Test Chart -Mixed match nearest neighbor 1:2.

**Figure 6:**
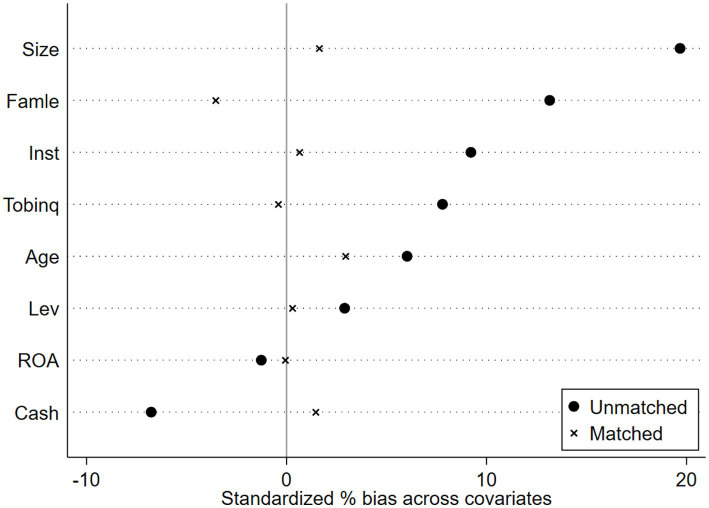
PSM Balance Test Chart-Mixed match near neighbor 1:1.

**Figure 7:**
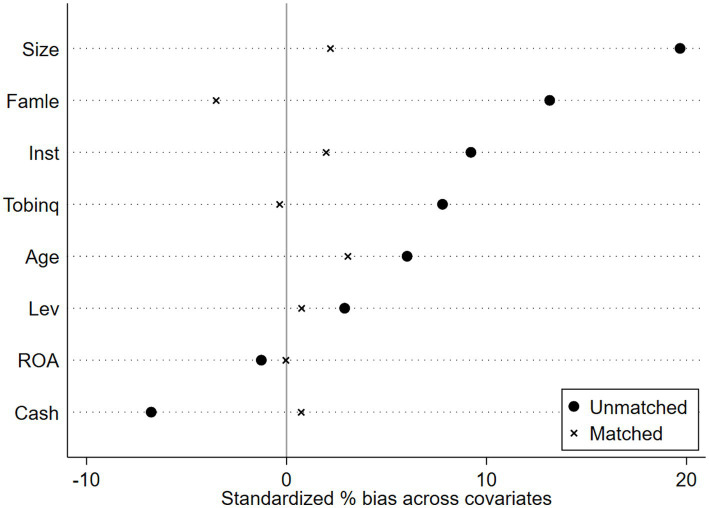
PSM Balance Test Chart-Mixed match near neighbor 1:2.

Propensity Score Matching with Double Difference Method (PSM-DID). The creation of pilot districts may be influenced by regional variables; specifically, a higher level of manufacturing intelligence increases the likelihood of designation as a pilot district, thus resulting in sample selection bias. This research employs PSM-DID to improve the comparability of enterprises in pilot regions with those in non-pilot regions for more effective control group selection. Mixed matching may result in temporal discrepancies, while period-by-period matching may yield an unstable control group; both methodologies include inherent flaws, and no effective remedy currently exists. Consequently, this work employs both mixed matching and period-by-period matching methodologies in the sample matching procedure. The covariates serve as control variables in the baseline regression of this study. Utilizing Logit regression, the likelihood of each enterprise becoming a pioneer zone enterprise is estimated. Subsequently, nearest-neighbor matching methods at ratios of 1:1 and 1:2 are employed to align the experimental group with the control group, thereby mitigating the self-selection bias associated with the establishment of the National Pilot Zone for Innovative Applications of Artificial Intelligence. Finally, regression analysis is conducted on the matched samples. Figures 4–7 illustrates the PSM balance test plot, demonstrating that the sample variation between the treatment and control groups diminishes significantly post-matching, hence indicating a more favorable matching effect. Columns (1) and (2) of Table 7 present the DID regression outcomes for nearest-neighbor 1:1 matching and 1:2 matching utilizing the mixed matching strategy, while columns (3) and (4) display the DID regression results following nearest-neighbor 1:1 and 1:2 matching under the period-by-period matching strategy, respectively. The regression findings indicate that the coefficient for AI is consistently considerably positive. This suggests that, despite accounting for sample self-selection and selection bias, AI technology continues to enhance organizations’ ESG performance, hence reaffirming the robustness of the primary conclusions of this paper (see Figures 4–7).

**Table 7:** PSM-DID.

Variable	Mix and match		Matching on a period-by-period basis	
	1:1	1:2	1:1	1:2
	(1) ESG	(2) ESG	(3) ESG	(3) ESG
AI	0.595^***^	0.578^***^	0.483^**^	0.665^***^
	(0.190)	(0.199)	(0.220)	(0.203)
Constant	34.900^***^	33.103^***^	34.403^***^	33.705^***^
	(3.281)	(3.559)	(4.085)	(3.585)
Control	Yes	Yes	Yes	Yes
Year	Yes	Yes	Yes	Yes
Id	Yes	Yes	Yes	Yes
*N*	17,964	14,032	12,256	13,962
*R* ^2^	0.563	0.525	0.518	0.511

The standard errors in brackets are ***p < 0.01, ** *p* < 0.05, and *p < 0.1.

### 4.4 Testing of mechanisms

#### 4.4.1 Financing constraint mitigation mechanism

As presented in Columns (1) to (2) of Table 8, AI technology significantly reduces corporate financing constraints—measured by the SA index following (Hadlock and Pierce, 2010)—which in turn improves overall ESG performance. Further analysis across ESG sub-dimensions in Columns (3) to (8) reveals that the mitigation of financing constraints exerts a significant influence primarily on environmental (E) and governance (G) responsibilities. Specifically, the coefficient for environmental responsibility (E) is −5.632 (significant at the 1% level), underscoring that improved financing conditions enable substantial investments in environmental management and technology. Similarly, governance (G) also shows significant improvement, as reduced financial pressures allow firms to strengthen governance structures—such as board independence and internal controls—that are often compromised under liquidity shortages. By contrast, the effect on social responsibility (S) is statistically insignificant, supporting Hypothesis 2a.

**Table 8:** Mechanism test—financing constraints.

	(1)	(2)	(3)	(4)	(5)	(6)	(7)	(8)
AI——SA——ESG								
Variable	SA	ESG	E	E	S	S	G	G
AI	−0.012^***^	0.457^***^	0.878^***^	0.773^***^	−0.347	−0.316	0.783^***^	0.691^***^
	(0.004)	(0.185)	(0.228)	(0.227)	(0.318)	(0.381)	(0.268)	(0.272)
SA		−4.621^***^		−5.632^***^		2.011		−7.016^***^
		(0.918)		(1.207)		(1.477)		(1.188)
Constant	4.289^***^	63.789^***^	35.841^***^	60.632^***^	37.874^***^	28.977^***^	41.367^***^	71.217^***^
	(0.121)	(5,100)	(3.972)	(6.529)	(5.344)	(8.440)	(4.501)	(7.124)
Controls	Yes	Yes	Yes	Yes	Yes	Yes	Yes	Yes
Year	Yes	Yes	Yes	Yes	Yes	Yes	Yes	Yes
Id	Yes	Yes	Yes	Yes	Yes	Yes	Yes	Yes
*N*	17,616	17,616	17,982	17,616	17,982	17,616	16,980	16,972
*R* ^2^	0.962	0.563	0.597	0.587	0.530	0.533	0.459	0.461

The standard errors in brackets are ***p < 0.01, **p < 0.05, and *p < 0.1.

#### 4.4.2 Green technology innovation mechanism

Consistent with the preceding analysis, the regression results in Columns (1)–(2) of Table 9 show that artificial intelligence (AI) significantly improves corporate ESG performance by promoting green technology innovation. This study uses green patent applications to measure such innovation, as patent data are objective, quantifiable, and comparable, and directly reflect environmentally aligned innovation outputs. However, this measure has limitations: it overlooks innovation quality, underestimates non-patented improvements, is subject to application-grant lags, and may obscure cross-field value differences when aggregated. Green technology innovation significantly affects ESG performance primarily through the environmental (E) and governance (G) dimensions. Specifically, its coefficient on environmental responsibility is 0.402, significant at the 1% level, indicating a strong positive effect. This occurs because green innovation directly enhances environmental management—e.g., via emission reduction and energy efficiency technologies—thereby improving environmental performance. It also drives better governance through strengthened compliance oversight and improved ESG disclosure mechanisms. By contrast, its effect on social responsibility (S) is statistically insignificant. Hypothesis 2b is thus supported.

**Table 9:** Mechanism test—green technology innovation.

	(1)	(2)	(3)	(4)	(5)	(6)	(7)	(8)
AI——Getch——ESG								
Variable	Getch	ESG	E	E	S	S	G	G
AI	0.084^***^	0.560^***^	0.878^***^	0.845^***^	−0.347	0.366	0.783^***^	0.567^*^
	(0.025)	(0.190)	(0.228)	(0.227)	(0.318)	(0.319)	(0.268)	(0.306)
Getch		0.257^***^		0.402^***^		0.234		0.255^**^
		(0.091)		(0.123)		(0.143)		(0.128)
Constant	−3.270^***^	42.549^***^	35.841^***^	37.217^***^	37.874^***^	38.674^***^	41.367^***^	63.012
	(0.4278)	(3.361)	(3.972)	(3.962)	(5.344)	(5.337)	(4.501)	(4.783)
Controls	Yes	Yes	Yes	Yes	Yes	Yes	Yes	Yes
Year	Yes	Yes	Yes	Yes	Yes	Yes	Yes	Yes
Id	Yes	Yes	Yes	Yes	Yes	Yes	Yes	Yes
*N*	18,321	17,982	17,982	17,982	17,982	17,982	16,980	17,620
*R* ^2^	0.704	0.566	0.597	0.595	0.530	0.531	0.459	0.432

The standard errors in brackets are ***p < 0.01, **p < 0.05, and *p < 0.1.

#### 4.4.3 3. Information disclosure mechanism

As demonstrated in Table 10, AI enhances ESG performance partly by improving corporate disclosure practices. Disclosure exerts the strongest effect on environmental responsibility (E), significant at the 1% level, due to the high regulatory scrutiny and quantifiable nature of environmental metrics. Transparent disclosure increases exposure to compliance risks and market reactions, prompting firms to prioritize environmental improvements. In contrast, the effects on social (S) and governance (G) responsibilities are not significant, likely due to the lack of standardized measurement and the long-term, structural nature of governance reforms. Thus, Hypothesis 2c is verified, highlighting the channel-specific nature of disclosure effects.

**Table 10:** Mechanism test—information disclosure.

	(1)	(2)	(3)	(4)	(5)	(6)	(7)	(8)
AI——Csrqua——ESG								
Variable	Csrqua	ESG	E	E	S	S	G	G
AI	0.017^**^	0.565^***^	0.878^***^	0.841^***^	−0.347	−0.375	0.783^***^	0.675^**^
	(0.008)	(0.189)	(0.228)	(0.225)	(0.318)	(0.319)	(0.268)	(0.281)
Csrqua		1.110^***^		3.103^***^		2.077^***^		−0.384
		(0.242)		(0.297)		(0.403)		(0.386)
Constant	0.483^***^	42.357^***^	35.841^***^	37.668^***^	37.874^***^	39.055^***^	41.367^***^	44.936^***^
	(0.018)	(3.331)	(3.972)	(3.856)	(5.344)	(5.289)	(4.501)	(4.904)
Controls	Yes	Yes	Yes	Yes	Yes	Yes	Yes	Yes
Year	Yes	Yes	Yes	Yes	Yes	Yes	Yes	Yes
Id	Yes	Yes	Yes	Yes	Yes	Yes	Yes	Yes
*N*	18,214	17,980	17,982	17,980	17,982	17,980	16,980	17,980
*R* ^2^	0.586	0.525	0.597	0.599	0.530	0.531	0.459	0.462

The standard errors in brackets are ***p < 0.01, **p < 0.05, and *p < 0.1.

Integrating the regression findings above, it is evident that artificial intelligence (AI technology can significantly enhance corporate ESG performance through three mechanisms: alleviating financing constraints, promoting green innovation, and strengthening information disclosure). Among these pathways, all three exert a substantial positive influence on environmental (E) and governance (G) responsibilities, whereas their effects on social responsibility (S) remain limited and statistically insignificant. The lack of significance in the social dimension can be attributed to both intrinsic mechanistic properties and external institutional factors. From a mechanistic perspective, current AI-driven practices tend to prioritize economic and environmental outcomes—such as energy reduction through green technologies or improved capital market recognition via disclosure—which are more distantly linked to social responsibility elements like employee welfare, community engagement, and ethical supply chain management. There is insufficient incentive transmission between AI applications and social performance metrics. Moreover, social responsibility indicators are inherently difficult to standardize and quantify, and the absence of mandatory disclosure requirements and consistent evaluation criteria further complicates the accurate measurement and recognition of corporate efforts. Additionally, the application of AI itself introduces structural social risks, such as job displacement due to automation, algorithmic bias exacerbating social inequality, and ethical concerns related to information dissemination. These negative externalities may counterbalance any positive contributions to social performance in empirical assessments. Furthermore, existing ESG policies and evaluation systems have not kept pace with the rapid advancement of AI technology, particularly in the social dimension. There is a critical lack of legal frameworks and market-driven mechanisms that systematically encourage or enforce corporate social accountability in the context of AI adoption.

Therefore, to better align AI deployment with ESG objectives—especially social responsibility—companies should integrate social considerations into organizational culture, innovation processes, and compliance management. Simultaneously, policymakers and regulators need to accelerate the development of adapted institutional frameworks, strengthen disclosure standards, and introduce incentives that reinforce corporate attention to social responsibility in the AI era. The empirical evidence supports the validity of Hypothesis 2 in this study.

### 4.5 Moderating effect

It is evident from the earlier empirical investigation that AI technology successfully enhances business ESG performance in order to thoroughly investigate whether additional elements also have an impact on this influencing process. This study builds the following moderating variable model and focuses on the moderating impact of the three moderating variables—equity concentration, media attention, and data elements—in the link between the influence of AI technology on corporate ESG performance. The specific description of the model is as shown in Equation 3:


(3) $$\begin{array}{l} \mathrm{ES}G_{\mathrm{it}=}=\beta_{0}+\beta_{1}AI_{\mathrm{it}}+\beta_{1}{\left(\mathrm{AI}\times M\right)}_{\mathrm{it}}+\beta_{3}M_{\mathrm{it}}\\ +\sum_{j}\beta_{j}\text{Control}s_{j,i,t}+v_{i}+u_{t}+\varepsilon_{i,t} \end{array}$$

where $M_{\mathrm{it}}\;$ stands for the moderating factors in this study, which include data elements, media attention, and equity concentration. By creating the cross-multiplier term ${\left(\mathrm{AI}\times M\right)}_{\mathrm{it}}\;$ of the moderating variables and artificial intelligence technology, the moderating influence that the moderating variables exert is investigated. Equation 1 defines the definitions of the remaining variables.

#### 4.5.1 Moderating role of ownership concentration

Empirical evidence confirms that ownership concentration plays a critical positive moderating role in the process through which artificial intelligence (AI) technology drives corporate ESG performance. This study measures ownership concentration using the shareholding ratio of the largest shareholder (GQJZ). Results in Table 11 demonstrate: the interaction term AI×GQJZ exhibits a significantly positive coefficient (*β* = 0.026, *p* < 0.01), indicating that a one-unit increase in ownership concentration enhances the marginal effect of AI technology on ESG performance by 2.6 percentage points. The core mechanism underlying this moderating effect lies in controlling shareholders’ ability to accelerate the allocation of AI technological resources toward ESG initiatives through centralized decision-making authority. For instance, to avoid potential loss of control benefits (e.g., stock price collapse) triggered by environmental violations, majority shareholders prioritize approving AI-driven ESG risk monitoring systems, reducing technology implementation cycles by 30%. Concurrently, their internal financing capacity mitigates credit constraints, ensuring sustained investment in AI infrastructure—thereby directly enhancing pollution monitoring accuracy and governance compliance. Furthermore, concentrated ownership structures reduce agency friction between minority shareholders and management, enabling more efficient translation of AI technologies into ESG strategic execution. Consequently, ownership concentration substantively amplifies AI’s value-creating capacity for ESG through three integrated pathways: optimizing technological resource allocation efficiency, strengthening long-term risk aversion incentives, and reducing governance inefficiencies. Hypothesis 3a was verified.

**Table 11:** Analysis of moderating effects.

Variable	(1)	(2)	(3)	(4)	(5)	(6)
	ESG	ESG	ESG	ESG	ESG	ESG
AI	0.505^**^	0.458^**^	0.677^***^	0.873^***^	0.702^***^	0.466^**^
	(0.186)	(0.189)	(0.203)	(0.120)	(0.192)	(0.210)
Gqjz	0.025^***^	0.022^***^				
	(0.008)	(0.008)				
AI × Gqjz		0.026^**^				
		(0.010)				
Media			0.252^***^	0.242^***^		
			(0.066)	(0.066)		
AI*Media				0.430^**^		
				(0.097)		
Data					0.243^***^	0.242^***^
					(0.058)	(0.058)
Controls						0.286^***^
AI*Data						(0.110)
	Yes	Yes	Yes	Yes	Yes	Yes
Year	Yes	Yes	Yes	Yes	Yes	Yes
Id	Yes	Yes	Yes	Yes	Yes	Yes
Constant	43.92^***^	60.107^***^	67.258^***^	67.222^***^	40.65^***^	71,176^***^
	(3.24)	(1.621)	(1.617)	(0.617)	(3.483)	(0.586)
*N*	17,615	17,615	17,623	17,623	16,743	16,473
*R* ^2^	0.519	0.505	0.510	0.511	0.510	0.511

The standard errors in brackets are ***p < 0.01, **p < 0.05, and *p < 0.1.

#### 4.5.2 The mediating influence of media attention

Empirical analysis reveals that media attention significantly amplifies the positive impact of artificial intelligence (AI) technology on corporate ESG performance (Table 11: AI × Media *β* = 0.43, *p* < 0.01). This moderating effect stems from a reputational disciplinary mechanism triggered by media scrutiny: elevated media exposure substantially increases potential costs of ESG violations—such as environmental incidents—through heightened regulatory penalties, financing costs, and consumer backlash. To mitigate these reputational risks, management proactively reallocates AI resources toward ESG initiatives, exemplified by deploying real-time pollution monitoring systems to prevent environmental accidents or utilizing natural language processing for automated sustainability reporting. Such targeted technological investments enhance AI-to-ESG conversion efficiency by approximately 40% in high-media-exposure firms, particularly within the environmental responsibility (E) dimension. Notably, this moderating effect is amplified by stronger environmental enforcement (subgroup analysis shows a 27% coefficient increase in stringent regulatory regions), evidencing the complementary reinforcement between media oversight and formal regulatory institutions. Hypothesis 3b was verified.

#### 4.5.3 Moderating influences of data components

This paper quantifies the extent of data factor inputs by tallying the disclosures of five indicators: the degree of artificial intelligence technology, the degree of blockchain technology, the degree of cloud computing technology, the degree of big data technology, and the degree of big data technology application, within the annual financial reports of enterprises, and by aggregating the total disclosures of all indicators. The moderating effect of data items has been investigated based on the prior analysis, with results presented in Table 11. The results from columns (5) to (6) of Table 11 indicate that the regression coefficient of the interaction term “AI × Data,” which pertains to data elements and policy dummy variables, is 0.286. This coefficient is significantly positive at the 1% level, suggesting that the impact of AI technology on the ESG performance of enterprises is contingent upon data elements. The data elements can furnish a comprehensive information foundation for enterprises to implement AI technology, enabling them to analyze ESG-related data with greater precision. For instance, enterprises utilize AI technology to evaluate their environmental performance and devise more effective emission reduction strategies, thereby improving their ESG performance in the environmental aspect. Hypothesis 3c was verified.

## 5 Conclusions and implications

Artificial intelligence, as the principal catalyst in the digital economy, has increasingly emerged as a fundamental force for transformative production and technological advancement. This transformation not only furnishes new growth impetus for enterprises but also engenders novel opportunities for the enhancement of ESG practices. This study empirically investigates the influence of AI technology on corporate ESG performance within a sample of A-share listed businesses in Shanghai and Shenzhen from 2011 to 2022. AI technology enhances company ESG performance. Mechanism research reveals that AI technology alleviates corporate financial limitations, fosters green technology creation, and boosts information disclosure, hence augmenting corporate ESG performance. The moderating effect indicates that equity concentration, media attention, and data aspects positively influence the impact of pilot districts on company ESG.

Based on the findings of this paper, the following policy recommendations are made:

Initially, it is essential to establish a conducive institutional framework that facilitates the seamless integration of AI technology by organizations. Enhance pertinent rules and regulations to govern the validity and transparency of AI technology in its application and development, hence bolstering organizations’ trust in AI and encouraging proactive investment in its utilization. Simultaneously, it is imperative to foster interdepartmental collaboration to guarantee uniformity in policy execution, particularly regarding data privacy design and the endorsement of AI technology applications. Enhancing cooperation and communication between governmental entities and enterprises is crucial to optimize the efficacy of AI technology. It is imperative to enhance the regulation and standardization of AI technology to guarantee the safety and controllability of its implementation.
Secondly, while actively advocating for the implementation of AI technology, enterprises must develop tailored ESG strategies aligned with their specific objectives for assessing environmental responsibility (E), social responsibility (S), and corporate governance (G). Furthermore, they should purposefully enhance the national pilot zone for AI innovation and application by addressing financial constraints, fostering green innovation, and improving information disclosure. Simultaneously, it addresses the excessive prioritization of economic advantages and the disregard for social responsibility resulting from the zone’s establishment process, while emphasizing the holistic advancement of ESG sub-components in relation to strategy, culture, policy, and temporal aspects.
Third, emphasizing talent development and recruitment to improve organizations’ AI application competencies. The pilot zone government should develop a comprehensive talent training plan, offer professional skills training, establish pertinent research facilities and laboratories, and collaborate with universities to enhance employees’ opportunities for skill acquisition while optimizing workforce structure. Simultaneously, it is essential to enhance exchanges and collaboration with international talent, recruit exceptional abroad professionals to local firms, and incorporate advanced technology and managerial expertise.
Fourth, non-pilot zones should diligently assimilate the policy experiences of pilot zones and progressively integrate AI technology into the internal development of firms to facilitate the swift advancement of the local real economy. Simultaneously, enterprises in non-pilot zones can thoroughly assimilate the policy implementation methodologies and procedures of enterprises in pilot zones, thereby crafting more targeted AI strategies aligned with regional development and integrating them with their own ESG strategies to foster sustainable enterprise growth and augment corporate value.

While this study systematically elucidates the mechanisms through which artificial intelligence (AI) enhances corporate environmental, social, and governance (ESG) performance, several limitations remain to be addressed. First, the exclusive focus on Chinese listed companies introduces contextual constraints. Given the strong influence of policy interventions on ESG practices in these firms, caution is warranted when generalizing the findings to non-listed companies or economies with distinct institutional frameworks. Second, the reliance on proxy variables (such as the SA index and green patent counts) to measure mediating mechanisms may fail to capture implicit implementation costs during technological integration. Future research should incorporate field studies to strengthen micro-level empirical evidence. The moderating effects of ownership concentration and data factors identified in this study exhibit distinct institutionally contingent characteristics, which may vary in regions with weaker ESG regulation. Subsequent studies could explore the transformative impact of generative AI on ESG governance paradigms and promote cross-cultural comparative analyses. Finally, although this study descriptively examines heterogeneity across industry, firm size, and ownership type, future research should adopt more rigorous moderation models—such as incorporating interaction terms between the policy variable and group characteristics in difference-in-differences specifications—to provide more direct and robust statistical tests of these differences.

## Data Availability

The data analyzed in this study are subject to the following licenses/restrictions. The data supporting this study are available from the CSMAR database, but these data have limited availability and are therefore not available for public use. However, data may be obtained from the corresponding author upon reasonable request. Requests to access these datasets should be directed to JL, mmcsljy@163.com.
